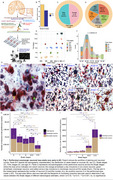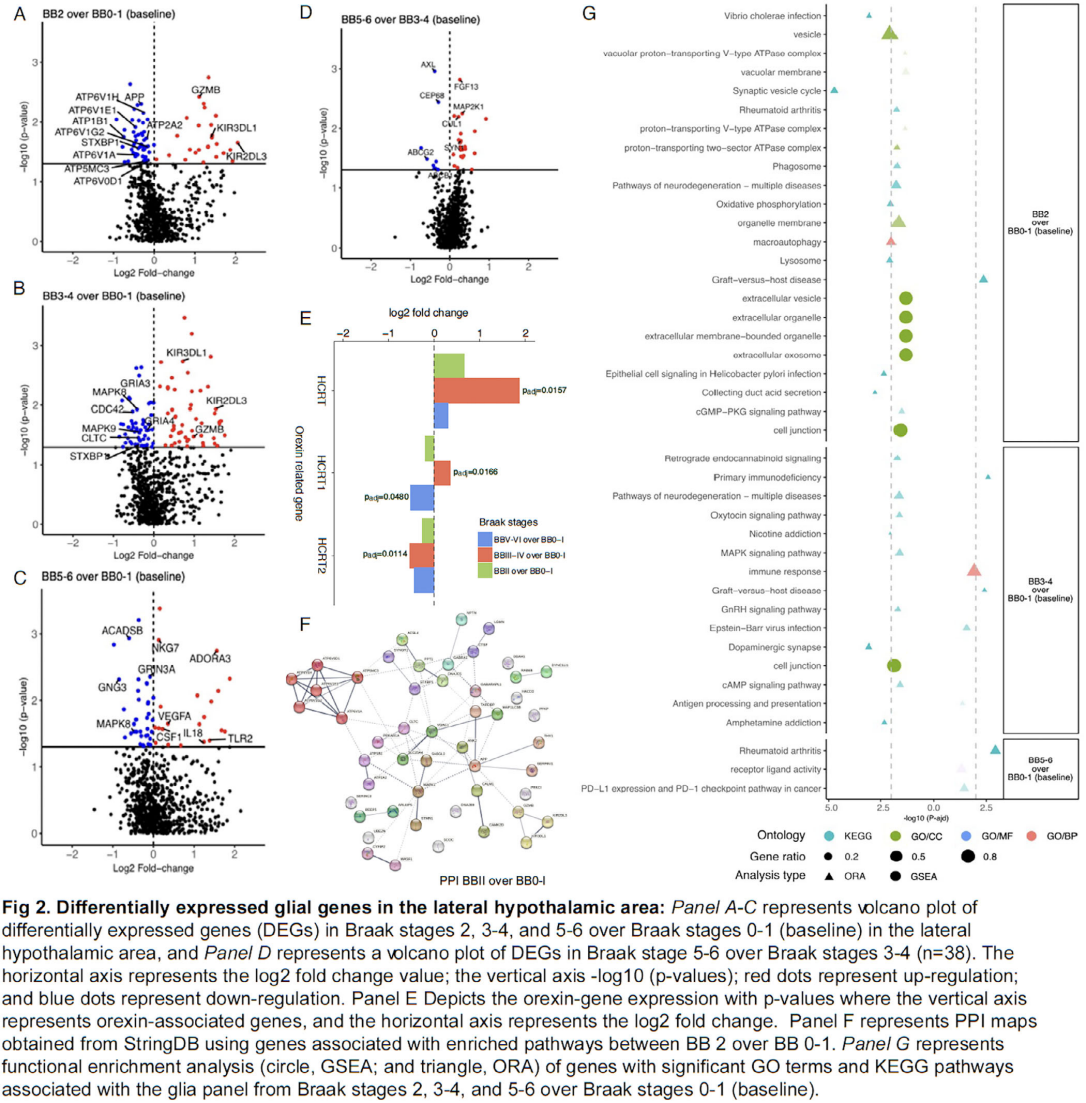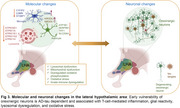# Focus on orexinergic neurons: omics study reveals glial changes and neuroinflammation in the lateral hypothalamic area as key factors driving the initial vulnerability in Alzheimer's disease

**DOI:** 10.1002/alz70855_107086

**Published:** 2025-12-24

**Authors:** Abhijit Satpati, Felipe Luiz Pereira, Alexander V. Soloviev, Mihovil Mladinov, Song Hua Li, Alexander Ehrenberg, Renata Elaine Paraizo Leite, Claudia Kimie Suemoto, Roberta Diehl Rodriguez, Vitor Ribeiro Paes, Christine M Walsh, Salvatore Spina, William W. Seeley, Carlos Augusto Pasquallucci, Wilson Jacob‐Filho, Thomas C. Neylan, Lea T. Grinberg

**Affiliations:** ^1^ Memory and Aging Center, UCSF Weill Institute for Neurosciences, University of California San Francisco, San Francisco, CA, USA; ^2^ Memory and Aging Center, UCSF Weill Institute for Neurosciences, University of California, San Francisco, San Francisco, CA, USA; ^3^ Innovative Genomics Institute, University of California, Berkeley, CA, USA; ^4^ Memory and Aging Center, University of California, San Francisco, CA, USA; ^5^ Physiopathology in Aging Laboratory (LIM‐22), Department of Internal Medicine, University of São Paulo Medical School, São Paulo, São Paulo, Brazil; ^6^ Division of Geriatrics, Department of Internal Medicine, University of São Paulo Medical School, São Paulo, São Paulo, Brazil; ^7^ University of São Paulo Medical School, São Paulo, São Paulo, Brazil; ^8^ Department of Neurology, Memory and Aging Center, University of California San Francisco, San Francisco, CA, USA; ^9^ Department of Psychiatry and Behavioral Sciences, University of California San Francisco, San Francisco, CA, USA; ^10^ Biobank for Aging Studies of the University of São Paulo, São Paulo, São Paulo, Brazil

## Abstract

**Background:**

The neuromodulatory subcortical system (NSS) is vital for homeostatic balance and is among the earliest regions to accumulate tau pathology in Alzheimer's disease (AD), undergoing marked degeneration by late‐stage AD. Within the NSS, orexinergic neurons (Orx^N^) regulate wakefulness, appetite, and sleep‐wake transitions. Experimental studies suggest that orexin dysfunction exacerbates AD, and recent clinical trials targeting the orexin pathway show improvements in AD biomarkers. However, the extent and timing of Orx^N^ loss in human AD patients, as well as the molecular mechanisms driving early Orx^N^ degeneration, remain unclear. We hypothesize that elucidating Orx^N^ vulnerability at early AD stages is essential for advancing orexin‐targeted therapies.

**Method:**

We used unbiased stereology on postmortem human brains across Braak stages 0–VI (*n* = 38). Hypothalamic sections were immunostained for orexin‐A and phosphorylated tau (CP13) antibodies, then counterstained with Nissl. We performed RNA sequencing of the lateral hypothalamic area (LHA) (*n* = 38), which houses all Orx^N^‐using the NanoString nCounter^®^platform, employing panels for neurotransmitter‐related genes, glial profiling, and neuropathology. Genes with padj<0.05 were deemed differentially expressed.

**Result:**

Stereological counts showed a 43% reduction in Orx^N^ at Braak I versus 0 (padj=0.0117), signifying the earliest massive neuronal loss in AD. After a plateau at Braak II‐IV, Orx^N^ declined by 70% at Braak V‐VI versus Braak 0 (padj=0.0062). A 50% drop in Orx^N^ at Braak II correlated with differential expression of genes related to lysosomal function, glial reactivity, oxidative stress, and phosphorylation. GO/KEGG analyses revealed enrichment of T‐cell‐mediated inflammatory, synaptic vesicle, and organelle dysfunction pathways. Despite extensive Orx^N^ loss, orexin‐related genes HCRT (padj=0.015) and HCRTR1 (padj=0.0166) were upregulated at Braak III‐IV.

**Conclusion:**

Orexinergic neurons are the earliest neuronal population to degenerate in AD, starting at Braak I. Early *p*‐tau accumulation in the LHA, independent of major β‐amyloid pathology, triggers T‐cell‐mediated inflammation, lysosomal dysregulation, and oxidative stress while surviving Orx^N^ attempts compensatory gene upregulation. Overall, this highlights Orx^N^ as a previously underrecognized target for early intervention, potentially offering both symptomatic and disease‐modifying benefits. Given the availability of orexin‐targeting pharmacotherapies, our data support expanded exploration of orexinergic modulation as a viable AD therapeutic strategy.